# A panel consisting of three novel circulating lncRNAs, is it a predictive tool for gastric cancer?

**DOI:** 10.1111/jcmm.13640

**Published:** 2018-04-26

**Authors:** Jingjing Liu, Jiajun Wang, Yongxi Song, Bin Ma, Junlong Luo, Zhongran Ni, Peng Gao, Jingxu Sun, Junhua Zhao, Xiaowan Chen, Zhenning Wang

**Affiliations:** ^1^ Department of Surgical Oncology and General Surgery First Hospital of China Medical University Shenyang China; ^2^ Department of Gastrointestinal Surgery The Second Hospital of Jilin University Changchun China

**Keywords:** biomarker, circulating lncRNA, gastric cancer, microarray, plasma

## Abstract

Early detection is vital for prolonging 5‐year survival for patients with gastric cancer (GC). Numerous studies indicate that circulating long non‐coding RNAs (lncRNAs) can be used to diagnose malignant tumours. This study aimed to investigate the capacity of novel lncRNAs for diagnosing GC. A lncRNA microarray assay was used to screen differentially expressed lncRNAs between plasma of patients with GC and healthy controls. Plasma samples from 100 patients with healthy controls were used to construct a multiple‐gene panel. An additional 50 pairs of GC patients with healthy controls were used to evaluate the diagnostic accuracy of the panel. Expression levels of lncRNAs were quantified through real‐time polymerase chain reaction. The receiver operating characteristic curve and area under curve (AUC) were used to estimate the diagnostic capacity. We identified three lncRNAs, CTC‐501O10.1, AC100830.4 and RP11‐210K20.5 that were up‐regulated in the plasma of GC patients with AUCs 0.724, 0.730 and 0.737, respectively (*P *<* *.01). Based on the logistic regression model, the combined AUC of the three lncRNAs was 0.764. The AUC of the panel was 0.700 in the validation cohort. These findings indicate that plasma lncRNAs can serve as potential biomarkers for detection of GC.

## 1 INTRODUCTION

Gastric cancer (GC) is the fourth most common malignant tumour worldwide, with its highest incidence rate in East Asia.^1^ According to the latest statistics, GC is the second most frequent cancer and accounts for the second most cancer‐related deaths in China, with a mortality of 498 000 per year.^2^ Many patients are diagnosed with GC already at an incurable late stage due to its non‐specific and easily‐neglected symptoms.^3^ Screening programmes in Korea and Japan have led to significantly increased 5‐year survival;^4^, ^5^ however, based on the large population, widespread screening in China presents many challenges.^6^ Despite having unsatisfactory sensitivity, carcinoembryonic antigen (CEA), carbohydrate antigen (CA) 199 and CA724 are still the most common serum markers used for GC detection in clinical practice. Thus, finding a new economic, non‐invasive and efficient diagnostic tool is critical for patients with GC.

Non‐coding RNAs (ncRNAs) comprise up to 75% of the entire human genome, and they perform many important regulatory roles in multiple biological processes.^7^ NcRNAs have been detected in many body fluids such as plasma, gastric juice, urine, and saliva.^8^ Researchers have also demonstrated that extracellular ncRNAs, including microRNAs (miRNAs) and long non‐coding RNAs (lncRNAs), can be used as diagnostic tools for conditions including heart failure,^9^ infectious diseases,^10^ type 2 diabetes^11^ and malignant tumours such as GC,^12^, ^13^ lung cancer^14^ and hepatocellular cancer.^15^ Examples include lncRNA SPRY4‐IT1 for diagnosis of hepatocellular carcinoma;^16^ miR‐22‐3p, miR‐642b‐3p and miR‐885‐5p for diagnosis of pancreatic cancer;^17^ and lncRNA MACC1 for diagnosis of non‐small‐cell lung cancer.^18^

Although many publications have demonstrated high accuracies and efficiencies for ncRNAs‐based diagnostic markers, the method is still controversial and far from clinical use due to the lack of large‐scale validation and consensus among researchers.^19^ In this study, with the aim to find novel biomarkers for GC, we detected dysregulated lncRNAs in patients with GC vs healthy controls based on the results of lncRNA microarray and investigated the diagnostic potential of plasma‐circulating lncRNAs.

## 2 MATERIALS AND METHODS

### 2.1 Study design, patients and plasma sample collections

A multiphase, case‐control study was conducted, and the whole study was divided into four phases: the discovery phase, testing phase, training phase and validation phase. In the discovery phase, lncRNA profiling analyses were conducted between plasma from four GC patients with age‐ and gender‐matched healthy controls. The top 15 differentially expressed lncRNAs were identified for further analysis in the next phase. In the testing phase, candidate lncRNAs were analysed in another 20 GC patients with matched healthy controls and in cell lines via real‐time polymerase chain reaction (PCR). Consistently, dysregulated lncRNAs were further investigated in the training phase, with 100 patients and 100 healthy controls. A combination of several lncRNAs was selected as a panel of GC diagnostic markers using a logistic regression model according to the real‐time PCR results. In the validation phase, the diagnostic values of the plasma lncRNAs were estimated in another 100 individuals (including GC patients with 50 and 50 healthy controls), and correlations between the lncRNA expression levels and the clinicopathological parameters of the patients were examined.

All enrolled patients with GC were diagnosed at The First Hospital of China Medical University (Shenyang, China) by histopathological examination after radical resection or endoscopic biopsy. Clinicopathological data were obtained from the medical records. Healthy controls consisted of patients with benign diseases such as hernias, haemorrhoids or varicose veins who had no evidence of any stomach disease or other malignancy. All clinical parameters were estimated according to the 8th AJCC/TNM staging system. Informed consent was obtained from all the participants, and this study was approved by the Research Ethics Committee of China Medical University.

Blood sampling was standardized. All blood samples were collected together with other blood‐based tests to minimize extra injury to the patient, and all were obtained after admission, under fasting conditions and before any treatment including operation or chemotherapy. Two millilitres of whole peripheral venous blood was collected from each enrolled individual and held in a purple‐top EDTA tube. Plasma samples were separated within 4 hours after collection following a two‐step centrifugation protocol (3000 *g* for 10 minutes at 4°C, 12 000 *g* for 10 minutes at 4°C) to thoroughly remove cellular nucleic acids, transferred to RNase/DNase‐free tubes and stored at −80°C until total RNA extraction.

### 2.2 Cell culture

Human GC cell lines SGC‐7901, MGC‐803, MKN‐45 and HGC‐27 and the normal gastric epithelial cell line GES‐1 were purchased from the Institute of Biochemistry and Cell Biology at the Chinese Academy of Sciences; Human GC cell lines KATO III and AGS were purchased from the American Type Cell Collection (ATCC). GC cell lines were cultured in RPMI 1640 medium (HyClone), and GES‐1 cells were cultured in Dulbecco's modified Eagle's medium (Invitrogen). All of the media contained 10% foetal bovine serum, and cells were kept in flasks at 37°C in a humidified atmosphere of 5% CO_2_. Every 2.0 × 10^6^ cells were seeded in 10 cm dishes with 5 mL culture media, and 48 hours after initial seeding, the culture media were collected and processed exactly as that described for plasma collection.

### 2.3 RNA extraction from plasma, cell lines and culture media

Total RNA was extracted from cultured cells using TRIzol reagent (Ambion) according to the manufacturer's protocol. Total RNA was extracted from plasma and culture media using the mirVana PARIS Kit (Ambion). 400 μL plasma or culture media were thoroughly mixed with an equal volume of 2 × denaturing solution and incubated on ice for 5 minutes 800 μL acid‐phenol:chloroform was added and thoroughly mixed via vortex, then centrifuged at 12 000 × *g* for 10 minutes at room temperature. The upper aqueous phase was transferred to a fresh RNase‐free tube. Washing steps were performed according to the manufacturer's protocol, and the RNA was finally eluted with 40 μL RNase‐free water pre‐heated to 95°C. Plasma samples for evaluating the internal control were randomly selected and processed under identical conditions.

### 2.4 Reverse transcription (RT) and real‐time PCR

Any residual DNA in the RNA sample was eliminated, and complementary DNA was synthesized using a PrimeScript® RT reagent kit (TaKaRa) according to the manufacturer's protocol. The relative expression levels of lncRNAs in all samples were determined using SYBR Premix Ex Taq II (TaKaRa) on a Light Cycler 480 II Real‐time PCR system (Roche). The reactions were incubated at 95°C for 30 seconds and then underwent 45 cycles of 95°C for 5 seconds and 60°C for 30 seconds. Samples were analysed in triplicate, and the products were confirmed by melting curve analysis following each reaction. Total cycles required for the SYBR signal to cross the threshold were identified as cycle threshold (Ct). Following the evaluation of the internal controls discussed in this study, the relative expression of each lncRNA in all samples was calculated using the Ct method normalized to β‐actin. All the human gene‐specific primers for real‐time PCR are shown in Table S1. Samples with a Ct > 40 were considered negative, as previously described.^20^

### 2.5 LncRNA microarray analysis

LncRNA microarray assays were performed by the Biotechnology Corporation using the SBC Agilent SurePrint G3 human lncRNA Microarray. Briefly, total RNA was extracted from the plasma samples of 4 patients with GC and matched healthy controls and purified using mirVana PARIS Kit (Ambion). RIN number was then checked to inspect RNA integrity by an Agilent Bioanalyzer 2100 (Agilent Technologies), and then RNA was amplified and transcribed into fluorescent cRNA using a Low Input Quick Amp Labeling Kit, One‐Color (Agilent Technologies). Labelled cRNA was purified using an RNeasy Mini Kit (Qiagen) and hybridized to the Agilent human lncRNA V6 Microarray (4 × 180K, platform: GPL21047, Agilent Technologies). Slides were scanned using an Agilent Microarray Scanner (Agilent Technologies), and data were extracted with Feature Extraction software 10.7 (Agilent Technologies). Raw data were normalized by quantile algorithm, limma packages in R.

### 2.6 Statistical analysis

Statistical analyses were performed by SPSS software, version 19.0, GraphPad Prism 5, and Stata version 12. The geNorm algorithm was used for evaluating the endogenous controls. The gene with the lowest stability value has the most stable expression. The geNorm default threshold value for stability is 1.5. ∆Ct is the difference between Ct values of the target and the endogenous reference β‐actin (∆Ct = Ct_lncRNA_‐Ct_β‐actin_). Student's *t* test was used to evaluate differences in expression of the chosen lncRNAs among the cell lines and between the plasma of the patients with GC and healthy controls. Receiver operating characteristic curve (ROC) and area under curve (AUC) were used to estimate the diagnostic value of each index for GC. The Youden index with corresponding sensitivity and specificity was calculated according to the formula: Youden index = maximum (sensitivity + specificity‐1) ≈ maximum (sensitivity‐[1‐specificity]). A combined ROC was calculated based on the logistic regression model. Pearson correlation coefficients were used to investigate similarity among the plasma‐based biomarkers. Two‐tailed *P* values <.05 were considered to be statistically significant.

## 3 RESULTS

### 3.1 General characteristics of the enrolled individuals

The median age of the patients with GC was 61 years (range, 29‐83 years), and the median age of the healthy controls was 60 years (range, 37‐89 years). A total of 108 patients with GC were male, and 42 were female. In the healthy controls group, 97 individuals were male, and 53 were female. There were no significant differences in age or gender between the two groups. Among the patients with GC, 26 of the 150 patients were diagnosed at an incurable stage and treated with chemotherapy or palliative operation.

### 3.2 Evaluation of candidate endogenous controls

There is currently no consensus on the use of endogenous controls for the relative quantification of circulating RNAs. Thus, we tested GAPDH, β‐actin, 18S RNA, RPL13, HPRT and PPIA as candidate endogenous controls, examining their expression in 16 plasma samples (8 samples from patients with GC, 8 samples from healthy controls). The stability value calculated by geNorm ranked β‐actin as the most stable endogenous reference (Raw Ct values for the candidate endogenous controls are shown in Table S2).

### 3.3 Discovery phase: Screening of candidate lncRNAs by microarray

**Figure 1:**
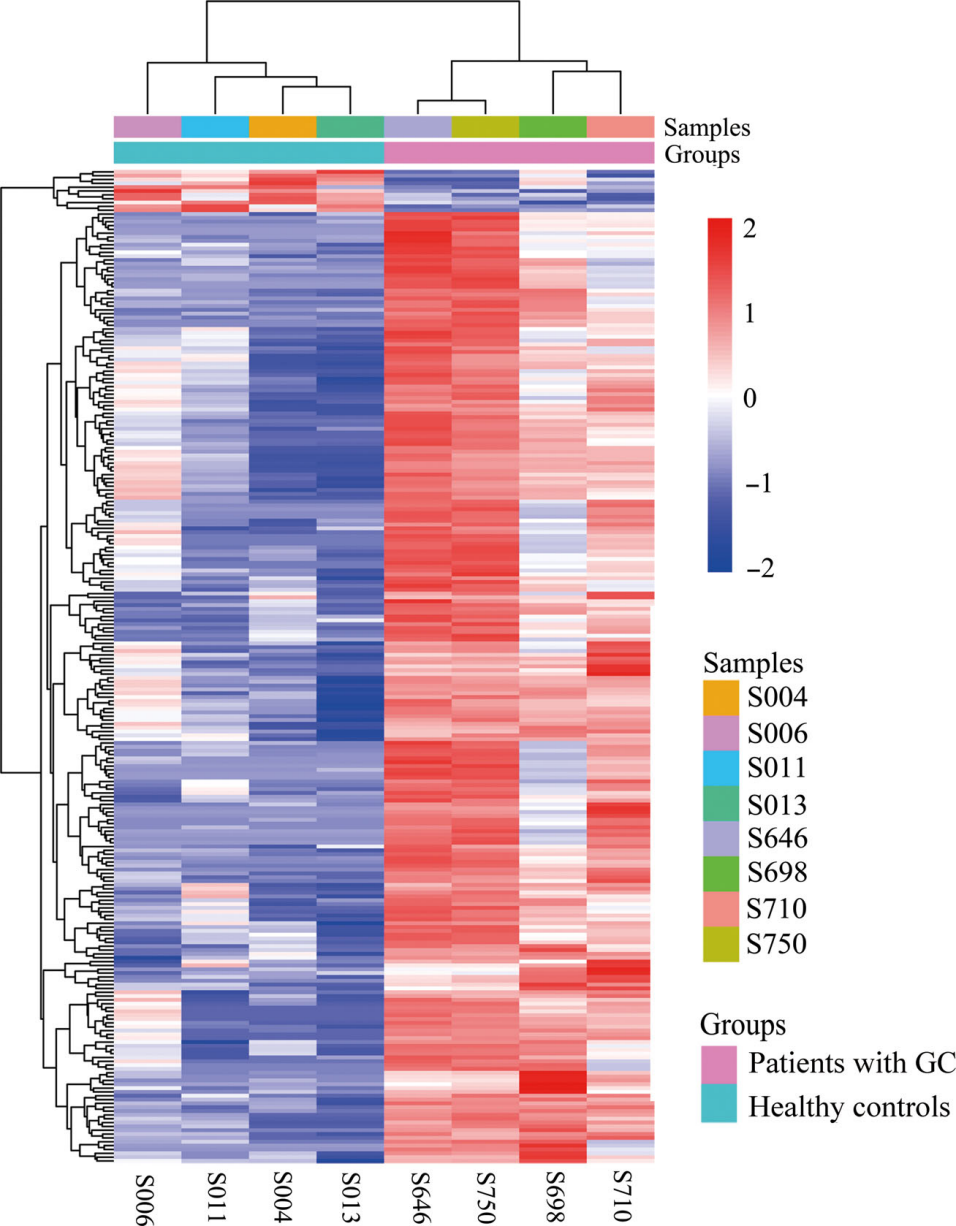
Heatmap result of microarray analysis. Differentially expressed lncRNAs (*P* < .05) between the plasma samples from 4 patients with gastric cancer and 4 healthy controls

The Human lncRNA V6 Microarray (Agilent) was used to screen for differential expression of lncRNAs in plasma samples from 4 patients with GC and 4 healthy controls. Fold change (GC vs Healthy) and *P* value were calculated from the normalized expression. Clustering analysis and heatmaps were used to show differentially expressed lncRNAs between the plasma samples from patients with GC and healthy controls (*P *<* *.05). We found that 267 lncRNAs were differentially expressed between these two groups. Among them, 256 lncRNAs were up‐regulated in the plasma of GC patients compared with healthy controls, and 11 lncRNAs were down‐regulated (Figure 1). The detailed lncRNA microarray data are shown in Table S3.

### 3.4 Testing phase: Analysing candidate lncRNAs in a small cohort and cell lines

We focused on the top 15 dysregulated results among the differentially expressed lncRNAs. These 15 lncRNAs were detected in 20 plasma samples from GC patients with 20 matched healthy controls via real‐time PCR. Three lncRNAs, CTC‐501O10.1, AC100830.4 and RP11‐210K20.5, were up‐regulated (Figure S1) and entered the next phase to perform large‐scale detection involving 200 plasma samples (100 samples for each group). The previously investigated lncRNAs H19 and HOTAIR were also included.^21^, ^22^ LncRNA H19 and HOTAIR were not significantly altered in a testing cohort (60 patients with GC vs 60 healthy controls, Figure S2).

**Figure 2:**
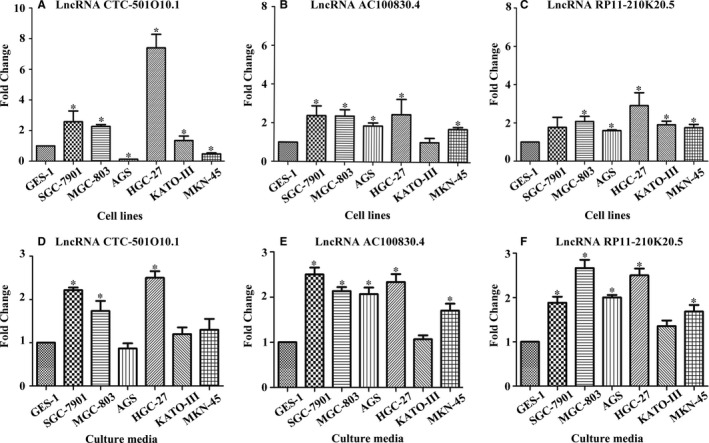
The relative expression levels of lncRNAs in gastric cancer cell lines and culture media. A‐C, Quantitative real‐time polymerase chain reaction (real‐time PCR) was used to measure the lncRNAs in gastric cells. β‐actin was used as normalization control. D‐F, The expression levels of the lncRNAs in culture media were measured using real‐time PCR. Data presented as fold change. Data are shown as mean ± SD, n = 3. *P < .05

Compared to normal gastric epithelial GES‐1 cells, we measured these three up‐regulated lncRNAs in multiple GC cell lines and culture media (SGC‐7901, MGC‐803, MKN‐45, HGC‐27, KATO III and AGS). CTC‐501O10.1 was up‐regulated in all GC cell lines except AGS and MKN‐45, AC100830.4 was up‐regulated in all GC cell lines except KATO III, and RP11‐210K20.5 was up‐regulated in all of the GC cell lines compared with GES‐1 cells. CTC‐501O10.1 was up‐regulated in SGC‐7901, MGC‐803 and HGC‐27 culture media. AC100830.4 and RP11‐210K20.5 were up‐regulated in SGC‐7901, MGC‐803, MKN‐45, HGC‐27 and AGS culture media. The relative expression level of each lncRNA is shown in Figure 2.

### 3.5 Training phase: Plasma expression of lncRNAs from patients with GC and healthy controls

**Figure 3:**
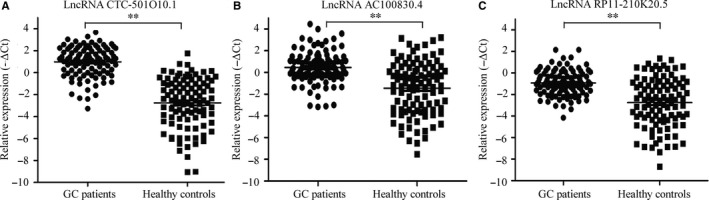
The relative expression levels of lncRNAs in plasma from patients with gastric cancer and healthy controls. A, The expression level of lncRNA CTC‐501O10.1 in plasma was higher in the gastric cancer group (*P *<* *.01). B, The expression level of lncRNA AC100830.4 in plasma was higher in the gastric cancer group (*P *<* *.01). C, The expression level of lncRNA RP11‐210K20.5 in plasma was higher in the gastric cancer group (*P *<* *.01)

We detected lncRNA CTC‐501O10.1, AC100830.4 and RP11‐210K20.5 in a total of 100 plasma samples from patients with GC and 100 plasma samples from gender‐ and age‐matched healthy controls. CTC‐501O10.1 was up‐regulated in plasma from GC patients with an average fold change of 3.41 (*P *<* *.01); AC100830.4 was up‐regulated in plasma from GC patients with an average fold change of 3.73 (*P *<* *.01); and RP11‐210K20.5 was up‐regulated with an average fold change of 3.56 (*P *<* *.01). The relative expression of each lncRNA is shown in Figure 3.

### 3.6 Training phase: Diagnostic potential of each lncRNA and establishment of diagnostic panel

**Figure 4:**
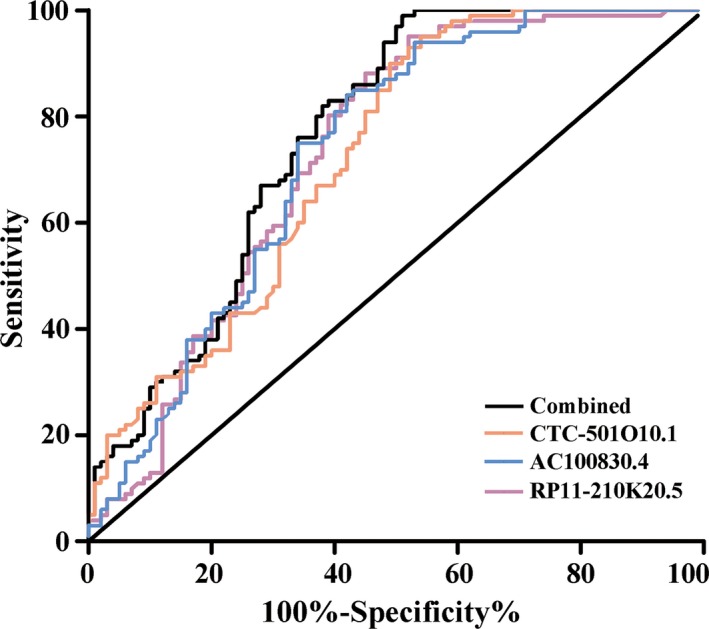
The AUC for each lncRNA in the training phase. The AUC for lncRNA CTC‐501O10.1, lncRNA AC100830.4 and lncRNA RP11‐210K20.5 was 0.724, 0.730 and 0.737, respectively. The combined AUC was 0.764 (*P *<* *.01)

The ROC and AUC were used to evaluate the potential of each lncRNA for GC detection. Relative expression levels of the lncRNAs were obtained via real‐time PCR. The AUC for CTC‐501O10.1 was 0.724 (95% potential, 0.654‐0.794, *P *<* *.01), the Youden index was 0.410, and the optimum sensitivity and specificity were 0.90 and 0.510, respectively. The AUC for AC100830.4 was 0.730 (95% potential, 0.659‐0.801, *P *<* *.01), the Youden index was 0.420, and the optimum sensitivity and specificity were 0.840 and 0.580, respectively. The AUC for RP11‐210K20.5 was 0.737 (95% potential, 0.666‐0.808, *P *<* *.01), the Youden index was 0.440, and the optimum sensitivity and specificity were 0.890 and 0.550, respectively. The combined ROC was drawn depending on the logistic regression results of the three lncRNAs, and the combined AUC was 0.764 (95% potential, 0.697‐0.831, *P *<* *.01) with a Youden index at 0.480, and optimum sensitivity and specificity of 0.990 and 0.490, respectively. The ROC for each index is shown in Figure 4.

### 3.7 Validation phase: Expression level and diagnostic potential of the lncRNA panel

**Figure 5:**
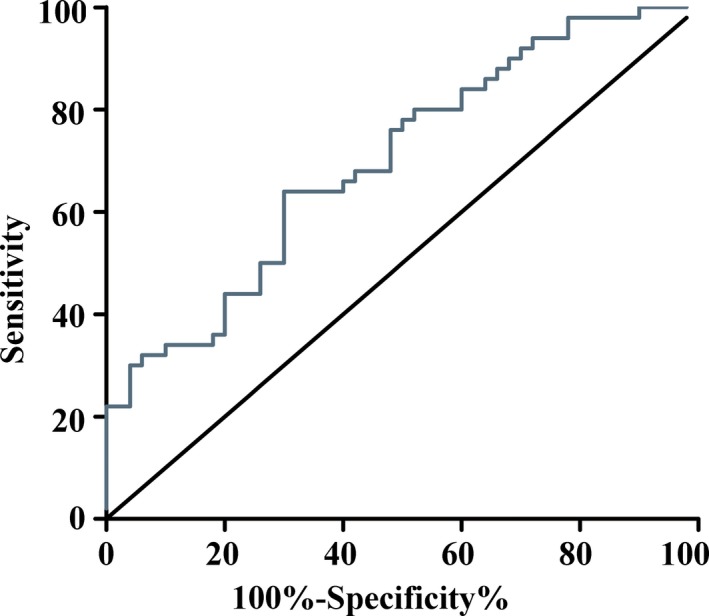
The AUC for the lncRNA panel in the validation phase. The AUC for the lncRNA panel was 0.700 (*P *<* *.01) in an independent cohort consisting of 50 patients with gastric cancer and 50 healthy controls

An independent cohort of 50 patients with GC and 50 healthy controls were used in the validation phase. The median age in both groups was 61 years. The results indicated that the panel could provide an AUC of 0.700 to distinguish GC from healthy controls based on the logistic regression model from the training phase (shown in Figure 5).

### 3.8 Correlation between the markers

To investigate the similarity in expression levels among the three lncRNAs, Pearson correlation coefficients were calculated for each marker. As presented in Table 1, the expression level of AC100830.4 showed a mutual relation with CTC‐501O10.1 with a coefficient of 0.463 (*P *<* *.01) and also mutually related with RP11‐210K20.5 with a coefficient of 0.268(*P *<* *.01).

**Table 1:** Pearson's correlation coefficients for the lncRNAs, CEA, AFP, CA125, CA153 and CA199

Biomarkers	CTC^a^	AC^b^	RP11^c^	CEA	AFP	CA125	CA153	CA199
CTC^a^	1	0.46**	0.13	0.03	−0.08	0.06	0.09	0.02
AC^b^		1	0.27**	−0.01	−0.03	−0.02	0.03	−0.02
RP11^c^			1	0.06	−0.07	0.12	0.11	0.16
CEA				1	−0.01	0.01	0.02	−0.01
AFP					1	0.03	0.08	0.02
CA125						1	0.39**	0.61**
CA153							1	0.25**
CA199								1

^**^Significant values (*P *<* *.01).

^a‐c^relative expression (−∆Ct) of CTC‐501O10.1, AC100830.4 and RP11‐210K20.5, respectively.

### 3.9 The correlation between expression level of lncRNAs and patient characteristics

We examined the correlations between the expression levels of circulating CTC‐501O10.1, AC100830.4 and RP11‐210K20.5 and patient clinicopathological parameters. No significant associations were observed between the lncRNAs and clinical parameters including age, gender, tumour location or tumour stage (Table S4).

## 4 DISCUSSION

In recent years, numerous studies have demonstrated that ncRNAs (lncRNAs, miRNAs and circular RNAs) are differentially expressed in cancer tissues and bodily fluids, that they often play regulatory roles in biological procedures, and that they have highly specific expression among different tissues.^23^, ^24^ It has been demonstrated that miRNAs and lncRNAs are both stable in the plasma, even in extreme conditions and also resistant to RNase A.^25^, ^26^, ^27^ These features make it plausible that ncRNAs could be used as diagnostic biomarkers for many types of cancer.^28^ For example, PCA3 is a significantly up‐regulated lncRNA in prostate cancer tissue and metastatic sites, and, based on a real‐time PCR assay, PCA3 is dysregulated in the urine of patients with prostate cancer. It has thus been reported to have clinical use as a diagnostic biomarker.^29^, ^30^

Because there is still no consensus on an endogenous reference for use with circulating RNAs, we evaluated many common choices and chose β‐actin for our endogenous control, consistent with other groups’ works.^20^, ^26^ In the current study, we performed a microarray to screen for deregulated lncRNAs in plasma from patients with GC and healthy controls and identified three novel lncRNAs, CTC‐501O10.1, AC100830.4 and RP11‐210K20.5, which were differentially expressed between these two groups with AUCs of 0.724, 0.730 and 0.737, respectively. The diagnostic potential seems unsatisfactory, yet it is still higher than that of CEA and CA199, as previously reported.^31^ The AUC of the single lncRNAs is similar to some previous studies.^32^

Due to the limited accuracy of any single index, several studies use multiple‐biomarker panels to elevate diagnostic accuracy, such as the miRNA panel established using miR‐16‐2, miR‐195, miR‐2861 and miR‐497 for cervical cancer detection.^33^ Likewise, a lncRNA panel consisting of lnc00152, CFLAR‐AS1 and POU3F3 was reportedly used for diagnosing oesophageal squamous cell carcinoma.^34^ The combination of three or more biomarkers can significantly elevate the diagnostic potential of blood‐based panels. For GC, some panels have been established, but none are widely used in clinical practice.^20^, ^35^ With the aim to increase diagnostic capacity, we combined three lncRNAs using a logistic regression model, resulting in an AUC of 0.764 in the training phase. The panel could provide a moderate diagnostic accuracy, with an AUC of 0.700 in the validation phase. Using the combination of these three lncRNAs, the diagnostic sensitivity was 0.99, which was meaningful in the clinic. To investigate why the panel could not provide a more optimal diagnostic potential, we analysed the correlation between the expression levels of lncRNAs and found that they were mutually related with each other (*P *<* *.05). The limited accuracy of the panel could be explained by the correlation in expression between the markers.^36^ The specific origins of circulating RNAs are largely unknown. It has been proposed that cancer tissues can secrete lncRNAs into the circulatory system and that they can be protected by nanoscale vesicles called exosomes. Thus, the correlation in expression may be due to the lncRNAs in bodily fluids arising from the same origin.^8^, ^37^ These circulating lncRNAs can be used as biomarkers and could even transfer drug‐resistance signals to other sites.^38^ CTC‐501O10.1 is located on chromosome 17 with a length of 549nt, AC100830.4 is located on Chr21 (q22, 12) with a length of 554nt, and RP11‐210K20.5 is located on Chr18 (q12, 1) with a length of 666nt. To the best of our knowledge, none of them have been investigated before. In this study, we found that CTC‐501O10.1, AC100830.4, and RP11‐210K20.5 could enter into the cell culture medium at a measurable level. And this provided evidence that lncRNAs were released into circulation and the dysregulation of the lncRNAs in circulation could be used as biomarkers for GC.

In our study, we found 3 novel dysregulated lncRNAs in plasma from patients with GC. Using these three lncRNAs, we could distinguish GC patients from healthy controls with better accuracy than some other biomarkers. It is not pessimistic when there was no significant association between the plasma lncRNAs and clinicopathological parameters. Many researchers reported that the lncRNAs in plasma showed no significant correlation with the clinic characteristics, such as some lncRNAs in clear cell renal cell carcinoma,^39^ in hepatocellular carcinoma^40^ and in gastric cancer.^20^, ^41^ The traditional biomarkers are the most common serum markers used for GC detection. Even in some studies, the traditional biomarkers such as CEA, CA19‐9 and CA724 showed no significant correlation with the clinic parameters.^42^ Although in the current study, the expression level of the lncRNAs is not correlated with any clinic parameters, but the diagnostic ability of the lncRNAs should not be neglected. They could provide better diagnostic ability than the traditional biomarkers, even better than the combination of CEA, CA19‐9 and CA724.^43^ Therefore, the circulation lncRNAs are potential diagnostic tools.

Interestingly, even the same lncRNA detected in different cohorts of patients may present different expression pattern. In our study, H19 and HOTAIR showed no significant differences between the plasma of patients with GC and healthy controls, even though it has been previously reported to be elevated in GC plasma.^20^, ^21^, ^44^ The current study is not the only one argued these discrepancies. H19 was once reported to be up‐regulated in plasma from patients with breast cancer;^45^ however at almost the meantime, another research reported that H19 was not altered in another cohort of patients with breast cancer.^46^ As to HOTAIR, it was also reported not altered in plasma from patients with GC in Otsuji's research, which was consistent with the current study.^41^ The inconsistency was not only seen in H19 and HOTAIR, but also in lncRNA MALAT1^20^, ^47^ and some miRNAs. For example, miR‐142‐3p and miR‐26a‐5p were previously reported to be down‐regulated in the plasma of patients with colorectal cancer, yet Malekzadeh reported that these two miRNAs were not altered in their plasma.^19^ Why the results of the same gene in different studies are so inconsistent? One reason may be the differences in the pathological status of the enrolled patients. Arita et al^41^ demonstrated that the inconsistency among the studies may be due to the heterogeneity or the primary tumour. There were more advanced‐stage patients enrolled in the studies of Zhang et al^21^ and Hashad et al,^44^ in which H19 and HOTAIR were up‐regulated. Especially, the stage IV patients accounted for 30%‐50% in these two studies. While in the current study, there were more early‐stage patients enrolled, and the stage IV patients accounted for 17% in the total number of the enrolled patients. Ke et al^48^ reported that lncRNAs exist in the plasma as fragments, and this hypothesis may provide another evidence for the inconsistency among the studies. In the hypothesis, different fragments have different expression levels, and different primers in these studies may be complementary with different fragments. They demonstrated that it is necessary to identify the most stable and dysregulated fragments as diagnostic biomarkers. However, this hypothesis is still controversial and needs further investigation. The inconsistency among the studies would be the biggest challenge before the clinic use of the plasma‐based‐RNA detection.

In summary, our results suggested that the dysregulated lncRNAs in plasma can be used as biomarkers for GC. Combined use of the lncRNAs could provide a higher diagnostic accuracy. However, further investigations and large‐scale case‐controlled studies are necessary to fully evaluate this novel option. Much work remains to find novel non‐invasive, economic and powerful biomarkers to detect diseases.

## CONFLICT OF INTEREST

The authors confirm that there are no conflict interests.
