# Supplementary figures and images for: A panel consisting of three novel circulating lncRNAs, is it a predictive tool for gastric cancer?
Source: J Cell Mol Med. 2018 Apr 26;22(7):3605–13. doi: 10.1111/jcmm.13640 (PMC6010868; doi:10.1111/jcmm.13640)

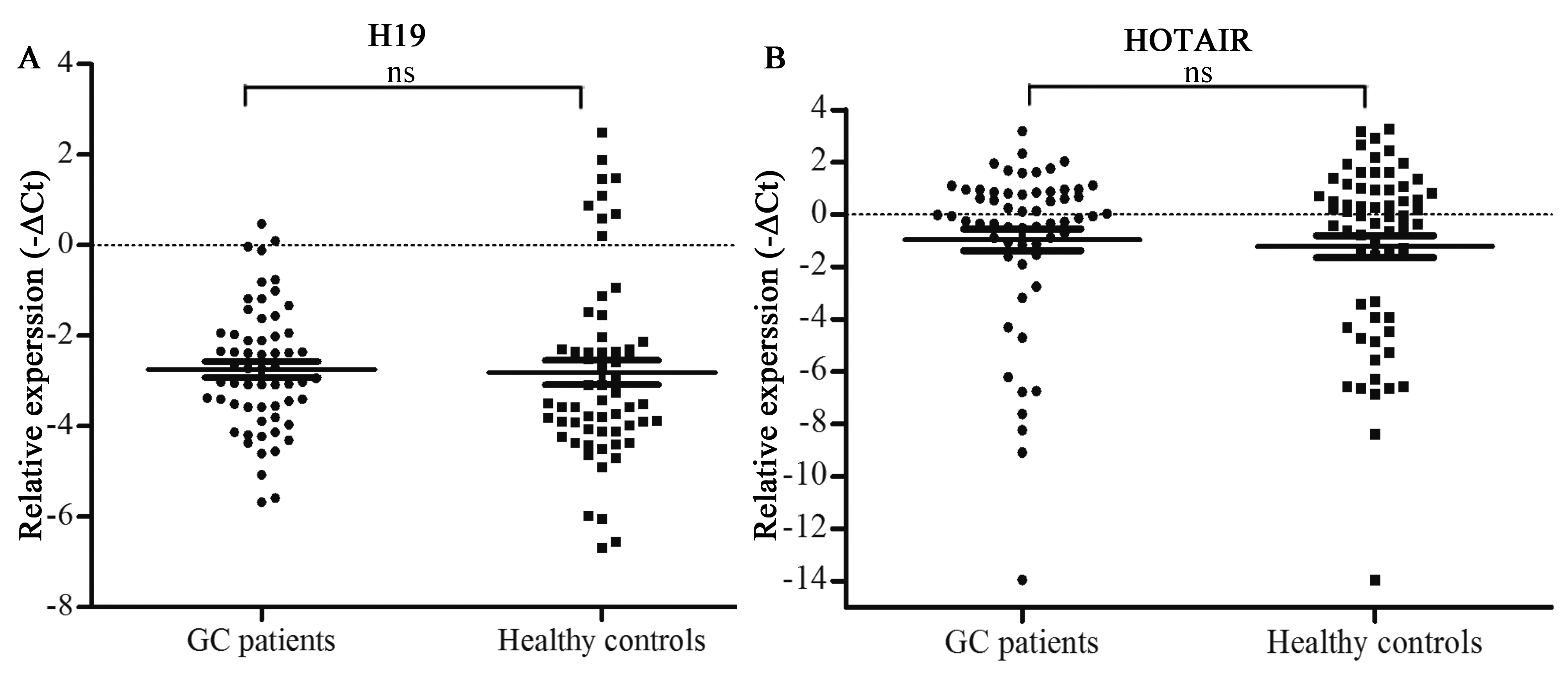

Supplement: Supplementary file 2 [file JCMM-22-3605-s002.tif]
