# Supplementary material for: A panel consisting of three novel circulating lncRNAs, is it a predictive tool for gastric cancer?
Source: J Cell Mol Med. 2018 Apr 26;22(7):3605–13. doi: 10.1111/jcmm.13640 (PMC6010868; doi:10.1111/jcmm.13640)
Supplement: Supplementary file 3 [file JCMM-22-3605-s003.docx]

| **Supplementary Table 1. Primers for real-time PCR** | | |
| --- | --- | --- |
|  | Forward primer | Reverse primer |
| 18s | TTGACGGAAGGGCACCACCAG | GCACCACCACCCACGGAATCG |
| GAPDH | ACCCACTCCTCCACCTTTGAC | TGTTGCTGTAGCCAAATTCGTT |
| β-actin | ATAGCACAGCCTGGATAGCAACGTAC | CACCTTCTACAATGAGCTGCGTGTG |
| HPRT1 | AATTATGGACAGGACTGAACGTCTTGCT | TCCAGCAGGTCAGCAAAGAATTTATAGC |
| PPIA | CCCACCGTGTTCTTCGACAT | CCAGTGCTCAGAGCACGAAA |
| RPL13 | CGGACCGTGCGAGGTAT | CACCATCCGCTTTTTCTTGTC |
| AC100830.4 | AGAAGCAATGGGCCAACTCA | ATTAGTGCTGGACACAGGGC |
| CTC-501O10.1 | GAAGATGCAGCCTGGGAGAG | CAGCTGCCATGGAGAAGTCA |
| RP11-210K20.5 | TGCGGCCTTTTTCCTTTTGC | TGACAAGTCAGGTTTTACAGCA |
| H19 | GCACCTTGGACATCTGGAGT | TTCTTTCCAGCCCTAGCTCA |
| HOTAIR | ACATTCTGCCC TGATTTCCG | CTTACCCC CACGGAGCAG |
