# Supplementary material for: A panel consisting of three novel circulating lncRNAs, is it a predictive tool for gastric cancer?
Source: J Cell Mol Med. 2018 Apr 26;22(7):3605–13. doi: 10.1111/jcmm.13640 (PMC6010868; doi:10.1111/jcmm.13640)
Supplement: Supplementary file 4 [file JCMM-22-3605-s004.docx]

| **Supplementary Table 2. The raw Ct values of the candidate endogenous controls** | | | | | | |
| --- | --- | --- | --- | --- | --- | --- |
|  | 18s | PPIA | HPRT1 | RPL13 | β-actin | GAPDH |
| GC01 | 26.59 | 34.58 | 36.48 | 32.03 | 33.08 | 35.45 |
| GC02 | 26.15 | 34.38 | 34.60 | 32.00 | 32.21 | 35.15 |
| GC03 | 26.92 | 35.52 | 35.39 | 32.75 | 32.83 | 35.77 |
| GC04 | 27.60 | 35.31 | 36.75 | 32.95 | 34.09 | 36.53 |
| GC05 | 26.65 | 34.81 | 39.83 | 31.63 | 32.01 | 35.11 |
| GC06 | 26.36 | 34.47 | 34.90 | 31.61 | 31.89 | 35.33 |
| GC07 | 27.26 | 35.68 | 36.57 | 32.90 | 33.53 | 35.32 |
| GC08 | 27.18 | 35.08 | 34.97 | 32.62 | 33.01 | 36.39 |
| H01 | 26.96 | 35.18 | 36.94 | 32.59 | 32.87 | 36.70 |
| H02 | 27.42 | 36.31 | 36.40 | 32.22 | 32.79 | 34.98 |
| H03 | 26.68 | 34.16 | 35.29 | 30.58 | 31.65 | 36.15 |
| H04 | 27.53 | 34.51 | 35.09 | 32.00 | 31.90 | 37.30 |
| H05 | 25.42 | 34.05 | 35.54 | 30.90 | 31.20 | 36.31 |
| H06 | 27.24 | 34.57 | 35.46 | 31.80 | 31.99 | 35.47 |
| H07 | 25.36 | 33.51 | 35.09 | 30.56 | 30.77 | 35.20 |
| H08 | 25.59 | 33.95 | 36.41 | 30.78 | 31.10 | 35.80 |
