# Supplementary material for: A panel consisting of three novel circulating lncRNAs, is it a predictive tool for gastric cancer?
Source: J Cell Mol Med. 2018 Apr 26;22(7):3605–13. doi: 10.1111/jcmm.13640 (PMC6010868; doi:10.1111/jcmm.13640)
Supplement: Supplementary file 6 [file JCMM-22-3605-s006.docx]

**Supplementary Table 3. The correlation between expression level^1^ of lncRNAs and patient characteristics^2^**

| **Clinical parameters** | **No.** | **CTC-501O10.1** | ***P*** | **AC100830.4** | ***P*** | **RP11-210K20.5** | ***P*** |
| --- | --- | --- | --- | --- | --- | --- | --- |
| **Gender** |  |  | 0.056 |  | 0.759 |  | 0.507 |
| Female | 42 | -1.437 |  | 0.349 |  | -0.123 |  |
| Male | 108 | -1.045 |  | 0.595 |  | -0.844 |  |
| **Age** |  |  | 0.461 |  | 0.645 |  | 0.408 |
| <61 | 65 | -1.103 |  | 0.757 |  | -0.735 |  |
| ≥61 | 85 | -1.290 |  | 0.470 |  | -0.993 |  |
| **Size^2^** |  |  | 0.476 |  | 0.691 |  | 0.588 |
| <4 cm | 60 | -1.337 |  | 0.507 |  | -1.013 |  |
| ≥4 cm | 64 | -1.252 |  | 0.215 |  | -1.118 |  |
| **Location** |  |  | 0.906 |  | 0.526 |  | 0.273 |
| U+M | 55 | -1.290 |  | 0.187 |  | -1.347 |  |
| L | 69 | -1.253 |  | 0.453 |  | -0.880 |  |
| **Differentiation** |  |  | 0.502 |  | 0.703 |  | 0.29 |
| Well + moderate | 29 | -0.995 |  | 0.308 |  | -1.173 |  |
| Poor + undifferentiated | 95 | -1.315 |  | 0.303 |  | -0.993 |  |
| **T category** |  |  | 0.939 |  | 0.922 |  | 0.79 |
| T1+T2 | 43 | -1.350 |  | 0.560 |  | -1.317 |  |
| T3+T4 | 81 | -1.250 |  | 0.225 |  | -0.992 |  |
| **N category** |  |  | 0.712 |  | 0.952 |  | 0.814 |
| N0 | 58 | -1.270 |  | 0.422 |  | -1.106 |  |
| N1-N3 | 66 | -1.279 |  | 0.264 |  | -0.993 |  |
| **AJCC stage** |  |  | 0.214 |  | 0.219 |  | 0.493 |
| I | 40 | -1.373 |  | 0.507 |  | -1.315 |  |
| II | 28 | -0.668 |  | 0.990 |  | -1.237 |  |
| III | 56 | -1.376 |  | 0.215 |  | -0.778 |  |

^1^Median of relative expression (-∆Ct); ^2^26 GC patients were diagnosed at an inoperable stage and excluded from the analysis of correlation between the expression levels of lncRNAs and the clinicopathological characteristics.
