# Supplementary material for: A panel consisting of three novel circulating lncRNAs, is it a predictive tool for gastric cancer?
Source: J Cell Mol Med. 2018 Apr 26;22(7):3605–13. doi: 10.1111/jcmm.13640 (PMC6010868; doi:10.1111/jcmm.13640)
Supplement: Supplementary file 7 [file JCMM-22-3605-s007.docx]

**Supplementary Figure 1.** Relative expression of lncRNAs in early testing phase.

**Supplementary Figure 2. The relative expression level of lncRNA H19 and HOTAIR in plasma samples.** A. The relative expression of H19 and there was no significant differences between the two groups (*P*>0.05). B. The relative expression of HOTAIR and there was no significant differences between the two groups (*P*>0.05).
